# Biometric Fingerprint System to Enable Rapid and Accurate Identification of Beneficiaries

**DOI:** 10.9745/GHSP-D-15-00010

**Published:** 2015-03-02

**Authors:** Daniel Matthew L Storisteanu, Toby L Norman, Alexandra Grigore, Tristram L Norman

**Affiliations:** a University of Cambridge, Department of Medicine, Cambridge, UK; b SimPrints Technology Limited, Cambridge, UK; c University of Cambridge, Judge Business School, Cambridge, UK; d University of Cambridge, Department of Chemical Engineering, Cambridge, UK; e University of London, The Royal Holloway, London, UK

## Abstract

Inability to uniquely identify clients impedes access to services and contributes to inefficiencies. Using a pocket-sized fingerprint scanner that wirelessly syncs with a health worker's smartphone, the SimPrints biometric system can link individuals' fingerprints to their health records. A pilot in Bangladesh will assess its potential.

With over 70% of births unregistered in least developed countries and 40% in developing countries, governments, multilateral health organizations, and NGOs increasingly recognize the “identification gap” as a major contributing factor to underdevelopment.^1^ Lack of official identity documentation, such as birth certificates, social security numbers, and medical records, obstructs people's access to basic rights and services.^1^^–^^4^ While health worker-driven mobile health (mHealth) programs are revolutionizing health care,^5^ challenges still exist in patient identification arising from lack of official identification, common community names, unknown dates of birth, and human error.

mHealth programs face challenges in patient identification due to lack of identity documentation.

Health workers may be responsible for tracking and monitoring the health of more than 1,000 individuals.^6^ The identification gap wastes resources (e.g., redundant vaccinations), limits information, excludes individuals from health services, and costs lives. Whereas in many places informal systems of identification have historically sufficed, increasing urbanization and migration pose growing identification challenges even for well-established health care providers.^2^ In order to tackle this issue, governments and NGOs are increasingly turning to biometrics.^2^^,^^7^^,^^8^

To help address the identification gap, we founded SimPrints, a nonprofit health technology organization centered on development of a pocket-sized fingerprint scanner that wirelessly syncs with a health worker's smartphone to link individuals' fingerprints to their health records (Figure 1).

**FIGURE 1.**
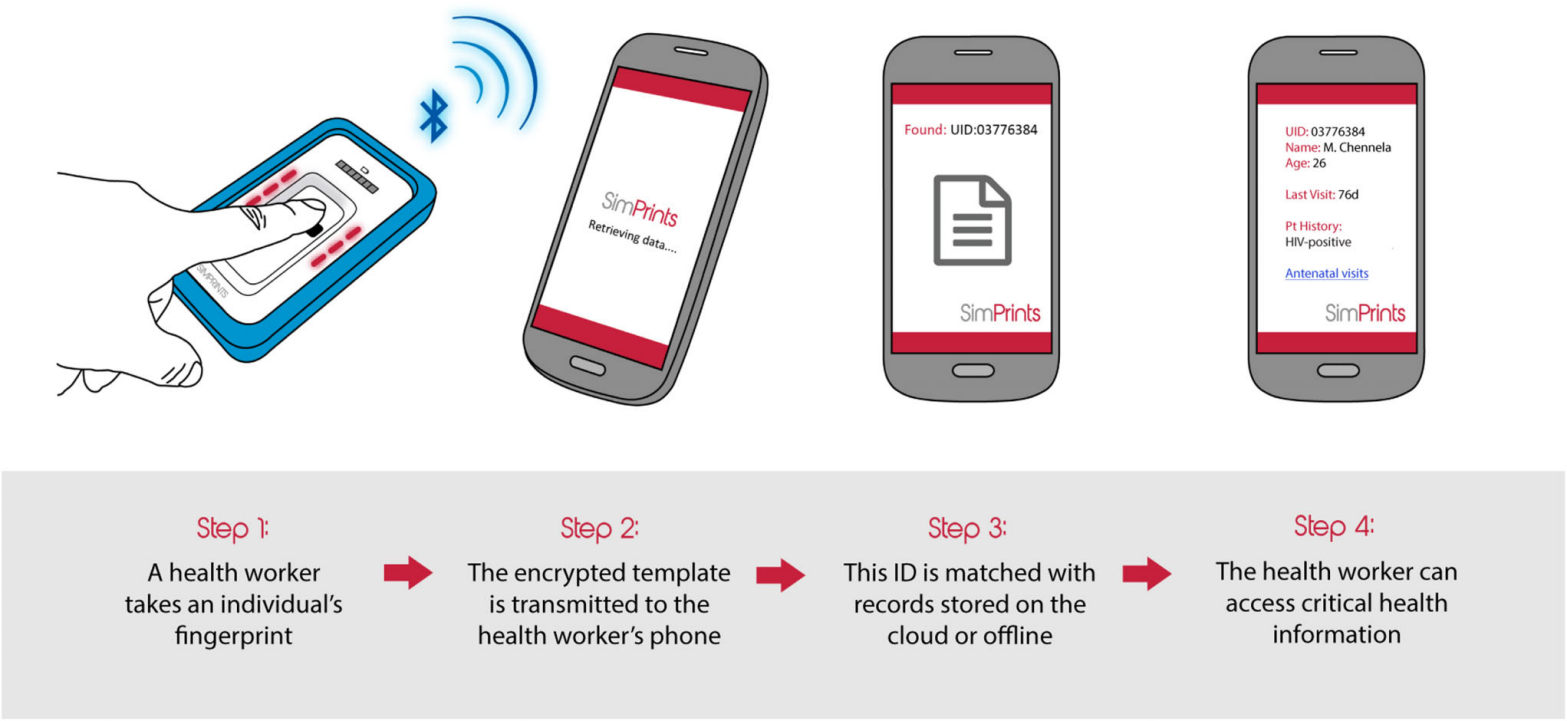
Use of the Simprints Biometric System by Health Workers

SimPrints is a nonprofit organization developing a small fingerprint scanner that syncs with a smartphone to link patients' fingerprints to their health records.

Use of fingerprint identification makes economic and practical sense: the sensors are accurate and inexpensive,^2^ and fingerprints are unique and cannot be lost. In order for the SimPrints system to function well in the field, we designed the hardware to be robust, low-cost, portable, and wireless. Its software will integrate with common mHealth platforms such as Open Data Kit (ODK) and CommCare, so that it can be easily used with most existing mHealth apps (Figure 2).

**FIGURE 2.**
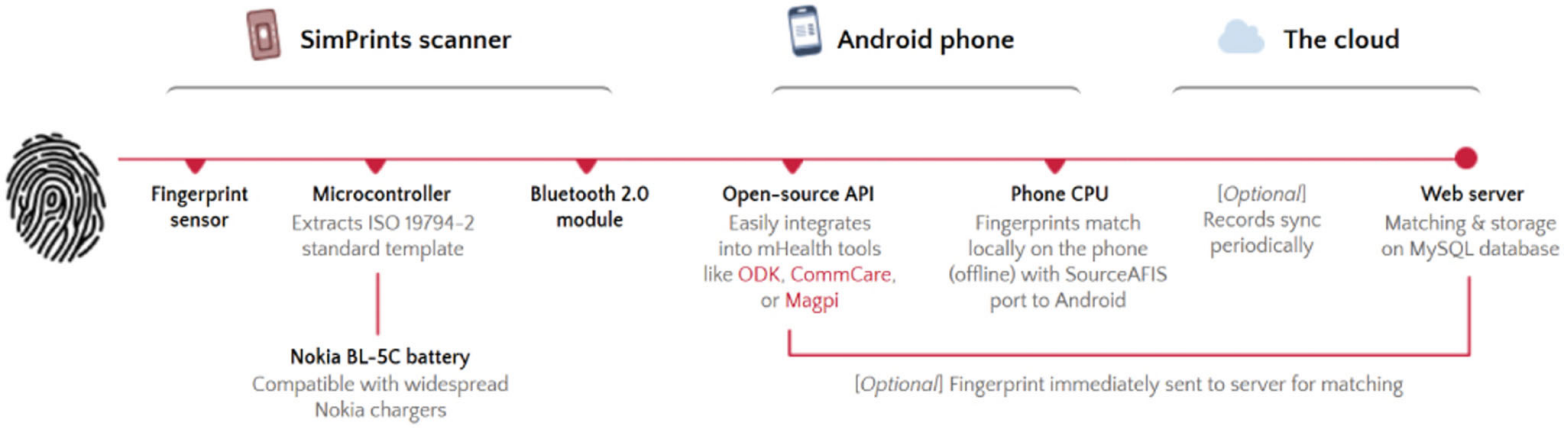
Technical Process Flow Diagram of the Simprints Biometric System

We envision this system enabling identification abilities in many of the most common mHealth and ICT applications,^9^ such as point-of-care diagnostics, data collection and reporting, registries/vital events tracking, supply chain management, and financial transactions and incentives. We plan to address privacy and security concerns by storing fingerprint data as encrypted templates instead of images, through database anonymization, and by using the same SSL/TLS (Secure Sockets Layer/Transport Layer Security) encryption standard used in online banking for data transfer and storage.

## BENEFITS OF THE SIMPRINTS SYSTEM

- High accuracy and secure identification
- Fast access and modification of records, allowing health workers in the field to make better decisions by providing immediate and reliable access to critical medical information
- De-duplication of records
- Enhanced privacy, as fingerprints do not convey personal details such as health status, gender, or race, and can be used in place of names
- Ensuring goods and services are reaching the intended beneficiaries
- Monitoring service delivery to enable the use of incentives for health workers, such as for successful prenatal visits
- Increasing program accountability by facilitating the measurement of indicators such as vaccination coverage
- Supporting civil registration and vital statistics systems by enabling tracking of vital events (e.g., births)

## POTENTIAL CHALLENGES

In areas where connectivity in the field is poor, the SimPrints system can access and modify offline health records that have been previously downloaded and are stored in a local database on the phone. Any updates to the health records will then be synced with the central database once Internet connectivity is restored. In order to increase access to charging points and make it easier to replace parts, the SimPrints scanner uses the same BL-5C Nokia batteries commonly used in mobile phones globally.

Challenges remain in fingerprint identification of infants, the elderly, and individuals with worn fingerprints due to manual labor. Strategies employed to prevent the exclusion of services to these individuals include connecting an infant's record to the fingerprints of their legal guardians and enrolling multiple fingerprints for manual laborers and the elderly to increase matching accuracy, as well as using secondary identification tags, such as their name or location, as a backup.

## NEXT STEPS

Supported by funding from the Saving Lives at Birth innovation grant and ARM Ltd., we will conduct a pilot study in partnership with BRAC and the Johns Hopkins Global mHealth Initiative to test the system with health workers in Gaibandha, Bangladesh. The study will focus on threshold testing to assess false positive, false negative, and failure-to-enroll rates, and research on performance analysis, usability, acceptability, usage patterns, and key health indicators such as the number of successful antenatal health visits.

A pilot study of the SimPrints system is planned with health workers in Bangladesh.

For more information about this biometric identification system, visit the SimPrints website at http://www.simprints.com.
